# Effect of formulations and adjuvants on the properties of acetamiprid solution and droplet deposition characteristics sprayed by UAV

**DOI:** 10.3389/fpls.2024.1441193

**Published:** 2024-08-02

**Authors:** Muhammad Zeeshan, Haoran Li, Gulfam Yousaf, Hao Ren, Yapeng Liu, Muhammad Arshad, Zechen Dou, Xiaoqiang Han

**Affiliations:** ^1^ Key Laboratory of Oasis Agricultural Pest Management and Plant Protection Resources Utilization, College of Agriculture, Shihezi University, Shihezi, China; ^2^ Department of Entomology, University of Sargodha, Sargodha, Pakistan

**Keywords:** formulations, adjuvants, physicochemical properties, droplet deposition, unmanned aerial vehicles

## Abstract

While the pesticide formulations are widely used for pest control, the combined effects of these formulations with adjuvants on droplet behavior, spraying characteristics, and pest control still need to be studied. To clarify their impact on droplet behavior, spraying characteristics, and control efficacy, six formulations of acetamiprid and six adjuvants were examined. A series of laboratory and field experiments were conducted to analyze the physicochemical properties, toxicity against cotton aphids, droplet deposition characteristics, and droplet drift. The results indicated that 5% acetamiprid micro-emulsion (ME) enhanced the physicochemical features and effectiveness in pest control compared to other formulations. The nongjianfei considerably enhanced the efficiency of all acetamiprid formulations when added. The addition of selected adjuvants to pesticide formulations improved the performance of certain physicochemical properties such as viscosity and surface tension and led to higher aphid mortality rates, demonstrating enhanced pest control effectiveness during the present study. In the field experiments, the combination effect of acetamiprid formulations and adjuvants exhibited a higher droplet size, coverage, and density within the cotton canopy. However, 5% acetamiprid ME was found to be most effective followed by nongjianfei. Furthermore, 5% acetamiprid ME with adjuvant reduced the droplet drift and provided better deposition when compared with other formulations. Overall, the combination of specific formulations and adjuvants led to improved physicochemical properties, enhanced droplet deposition characteristics, reduced spray drift, and increased pesticide deposition. These findings highlighted the significance of selecting appropriate pesticide formulations and adjuvants and provided a solid foundation for efficient pesticide spraying through UAVs.

## 1 Introduction

Cotton (*Gossypium* spp.) is the primary industry of the Xinjiang Uygur Autonomous Region, China, which plays a crucial role in the economic and social progress of the region (Lou et al., 2018). Contributing to nearly 30% of worldwide production, China stands as a dominant player in global cotton production (Dai et al., 2017). Cotton is a significant cash crop, found to be the largest production base in China’s Xinjiang Region, which accounts for 52% of the country’s output (Zhu et al., 2023). This industry has notably enhanced the local economy by improving the income of Xinjiang’s residents, becoming a pillar for them (Niu et al., 2021). In 2023, Xinjiang’s cotton planting area and yield constituted 84.98% and 90.99% of China’s total, respectively (National Bureau of Statistics, 2023). The biotic stressors induced by pests and diseases are widely recognized for their detrimental impact on world food grain supply, leading to significant losses. The cotton aphid, *Aphis gossypii* Glover (Homoptera: Aphididae), is a pervasive pest that infests a broad spectrum of vegetable and field crops globally. The detrimental impact of aphid infestation, which includes direct feeding, honeydew excretion, and virus transmission, results in substantial yield loss. Therefore, enhancing phytochemical prevention and control measures for cotton is crucial for improving both its yield and quality. The control of cotton aphids primarily relies on the use of various classes of insecticides, with neonicotinoids being widely adopted as alternatives to traditional insecticides for combating sucking insects (Mokbel, 2018).

Acetamiprid is a newly developed neonicotinoid insecticide that exhibits significant systemic and translaminar activity. It provides exceptional effectiveness against sucking pests such as leafhoppers, aphids, thrips, and whiteflies; these pests are widely recognized as highly destructive to agricultural crops worldwide (Bhamare, 2018). Acetamiprid against Homoptera, Thysanoptera, and Lepidoptera was found to be highly effective. Hence, it serves as a viable alternative for addressing the issue of resistant pest populations (Ijaz et al., 2016). Acetamiprid was chosen because of its widespread use, effectiveness against a wide range of sucking pests specifically cotton aphids, low toxicity against natural enemies and mammals, and lower environmental impact (Zuščíková et al., 2023).

In Xinjiang, the majority of cotton pesticide operations are conducted utilizing large-scale ground machinery. This practice leads to numerous mechanical injuries to the cotton crop, such as hitting the bolls, rolling the plant, pulling the branch, and knocking off opened balls. Hence traditional manual and semi-mechanical methods of plant protection not only result in excessive spraying and low pesticide utilization rates but also lead to substantial pesticide residues. With the rural labor population increasingly migrating to urban areas and the intensification of population aging, there is an immediate need for innovative pesticide application equipment that can accommodate small plots in hilly and mountainous regions (Wang et al., 2019). In recent years, the utilization of unmanned aerial vehicles (UAVs) for pesticide spraying in plant protection has experienced significant advancements, resulting in enhanced effectiveness of plant protection methods. As of 2023, the number of plant protection UAVs in China has exceeded 200,000, and the operating area has exceeded 2.1 billion acres (Ministry of Agriculture and Rural Affairs of the People's Republic of China, 2024). Despite these advancements, improper operation and incorrect pesticide spraying by agricultural operators have led to an excessive application of pesticides, resulting in an annual waste of 4.1 million tons worldwide (Liu et al., 2021).

In recent years, there have been significant studies conducted on spraying systems and of UAVs. Wang et al. (2020b) developed an electrostatic spraying system with bipolar contact for UAVs. This innovative system generates charged droplets that effectively coat the lower surfaces of leaves, significantly improving the adhesion of the droplets. As for UAV spraying performance, comprehensive studies have been conducted on droplet distribution and pesticide control effects. Early tests indicated uneven droplet distribution, considerable drift potential, and tube or nozzle blockage during UAV pesticide delivery (Brown and Giles, 2018; Wang et al., 2018a). Researchers have extensively studied the application settings of UAVs to enhance the deposition and drift characteristics of pesticide droplets. Wang et al. (2020a) explored the correlation between UAV spraying volumes and the deposition and efficacy of droplets, leading to the determination of an optimal spraying volume for improved control efficacy. Li et al. (2021) compared UAV technology with a conventional air blast sprayer, providing valuable data to enhance crop protection programs for large canopy crops using UAVs. Zheng et al. (2017) executed field trials, manipulating the flight variables of multi-rotor UAVs, to assess the impact of spraying during various growth phases of corn, supplemented by quantitative modeling. Wang et al. (2017) conducted field experiments with four representative UAVs to evaluate aspects such as droplet deposition, coverage rate, droplet density, penetration, and operational efficiency. Wang et al. (2024) studied the effect of adjuvants on pesticide performance and found that incorporating the adjuvants to the pesticide increased the droplet size, reduced droplet drift, and improved both the uniformity of deposition and penetration capabilities. Sun et al. (2024) explored the influence of various adjuvant types and their concentrations on the efficacy of suspension concentrates (SCs) when applied via UAVs and found that appropriate adjuvants and their optimal concentrations can significantly improve the delivery of pesticide dosages in conjunction with the formulation.

UAVs are currently employed extensively for pest and disease control in East Asia (Hunter et al., 2020). Despite their widespread use, UAVs’ aerial applications are often plagued by significant drift behavior, posing substantial safety risks to the surrounding environment and sensitive crops. The deposition and retention of pesticide spray on the surface of plant leaves are faced with major challenges (Topping et al., 2020). Consequently, incorporating an adjuvant into the pesticide solution is essential for improved application. These adjuvants modify the pesticide’s action and alter the physicochemical properties (Meng et al., 2021). Primarily, spraying adjuvants enhance the droplet deposition and retention on target plant leaves by reducing surface tension and increasing viscosity (Cao et al., 2018; Huang et al., 2020).

Factors such as evaporation, drift, and bouncing of spray droplets can contribute to pesticide loss, diminished efficacy, and heightened environmental risks. The efficacy of pesticide application is closely linked to the droplet wetting and spreading on the leaf surface, with droplet retention, surface tension, and contact angle being key influencers (Guo et al., 2019; Duan et al., 2022). Prior research has primarily focused on enhancing operational quality through UAV component improvements to boost pesticide efficiency (Zhao et al., 2022b). In particular, the use of conventional formulations in tandem with tank-mix adjuvants presents a more economical and convenient approach. However, there is very limited research on the use of formulations specifically acetamiprid and adjuvants on the physicochemical properties of solution for UAV-based plant protection. Additionally, there is a scarcity of systematic studies based on field experiments investigating droplet drift and deposition characteristics, coupled with the effectiveness of pest control in practical applications.

Recently, the advancements in UAV technology have brought a crucial shift in precision agricultural practices and offered a wide range of opportunities for efficient and targeted pesticide delivery (Taseer and Han, 2024). However, there is very little research on the aerial application of acetamiprid through UAVs. Additionally, there are very few or no reports available in the previous studies on the impact of various formulations of acetamiprid on the physicochemical properties, pest control efficacy, and droplet deposition characteristics particularly when combined with adjuvants. Furthermore, the use of various formulations and adjuvants stems from the need to optimize the application process of UAV spraying technology. In the present study, six formulations of acetamiprid and six adjuvants were examined. The study was focused to investigate the physicochemical properties of the formulations of acetamiprid solution in combination with adjuvants, the impact on pesticide deposition, and pest control efficacy. Moreover, we focused on analyzing the spray droplet characteristics, deposition, and drift of the liquid solution in order to enhance the pesticide efficacy and deposition to the targeted surface. By examining these variations, we aimed to identify the most effective combination that enhances deposition, reduces drift, and improves pest control outcomes.

## 2 Materials and methods

The experiment was conducted in two stages. The first stage was conducted in the laboratory while focusing on the physicochemical properties of the acetamiprid and adjuvant solution. These measurements included density, viscosity, surface tension, contact angle, spreading rate, and the efficacy of the solutions against cotton aphids. The second stage of the experiment was carried out in the field where the efficacy of the formulations and adjuvants was tested using UAV. The field experiment included the measurement of droplet size, coverage, droplet density, deposition, and droplet drift under various environmental conditions.

### 2.1 Insecticide and adjuvants

In this research, six different formulations of acetamiprid, water-soluble powder (SP), soluble concentrate (SL), emulsifiable concentrate (EC), water dispersible granule (WG), wettable powder (WP), and micro-emulsion (ME), and six different adjuvants, silwet 710, beidatong, hongyuyan, U partner, guoguang, and nongjianfei, were selected (**Tables 1**, **2**). Each formulation of acetamiprid was combined with every adjuvant to check the physicochemical properties of the solution. A total of 49 treatments were formed by combining the given formulations and adjuvants and water as control.

**Table 1:** Details of acetamiprid formulations used in the experiment.

Treatments	Pesticide name	Manufacturer	Recommended dose (ha^−1^)	Dosage of active ingredients (ha^−1^)
1	20% acetamiprid SP	Shandong Wanhao Chemical Co., Ltd., Dongying, China	150 g	30 g
2	20% acetamiprid SL	Qingdao Odis Biotechnology Co., Ltd., Qingdao, China	150 mL	
3	10% acetamiprid EC	Cangzhou Runde Pesticide Co., Ltd., Cangzhou, China	300 mL	
4	40% acetamiprid WG	Chengdu Kelilong Biochemical Co., Ltd., Chengdu, China	75 g	
5	20% acetamiprid WP	Shandong Sino-Agri Biotechnology Co., Ltd., Jinan, China	150 g	
6	5% acetamiprid ME	Liufuding Crop Protection Co., Ltd., Zhengzhou, China	600 mL	

**Table 2:** Details of adjuvants used in the experiment.

Treatments	Adjuvants	Type/Efficacy	Manufacturer	Dosage	v/v
1	Silwet710	Organosilicone/Moisturizing and spreading	Momentive (Shanghai) Trading Co., Ltd., Shanghai, China	2.5 mL/1.5 L	1/600
2	Beidatong	Vegetable oil/Anti-drift and evaporation	Hebei Mingshun Agricultural Technology Co., Ltd., Shijiazhuang, China	30 mL/1.5 L	1/50
3	Hongyuyan	High molecular polymer/Anti drift and evaporation	Shenzhen Noposion International Investment Co., Ltd., Shenzhen, China	20 mL/1.5 L	1/75
4	U partner	High molecular polymer/Anti-drift and evaporation	Beijing Grand AgroChem Co., Ltd., Beijing, China	20 mL/1.5 L	1/75
5	Guoguang	Mineral oil/Anti-drift and evaporation	Sichuan Runer Technology Co., Ltd., Jianyang, China	4 mL/1.5 L	1/375
6	Nongjianfei	High molecular polymer/Anti-drift and evaporation	Guilin Jiqi Group Co., Ltd., Guilin, China	8 mL/1.5 L	1/187.5

### 2.2 Physicochemical properties of acetamiprid solution

#### 2.2.1 Density

The density of the solution was determined using the weight loss method at room temperature (Duan et al., 2022). The process was repeated three times to ensure accuracy. The density of the solution was then obtained by using Equation 1.


(1) $$\text{ρ}\;=\;\frac{{\text{m}}_{2}-{\text{m}}_{1}}{25}$$

where *m*_1_ represents the weight (g) of a pycnometer flask with a capacity of 25 mL and *m*_2_ represents the weight (g) of the pycnometer flask after the test solution has been added.

#### 2.2.2 Viscosity

The viscosity of the liquid was determined using a pins capillary viscometer. A small-mouthed rubber ball (ear washing ball) was employed to connect the small glass tube to the fine tube. At room temperature, 10 mL of the sample solution was sucked into the second circle of the viscometer, which was then stood upright. The outflow time between the first and second laps was recorded. This process was repeated five times, ensuring a time difference of no more than 0.3 s (Liu et al., 2022). Viscosity was calculated according to Equation 2.


(2) η = vk × 0.00947

where η represents the dynamic viscosity (mPa·s), v_k_ represents the kinematic viscosity, and 0.00947 is the instrument constant for viscometer (mm^2^/s^2^) provided by the manufacturer.

#### 2.2.3 Surface tension

The surface tension was determined with the help of a completely automatic surface/interfacial tension meter (Kino A60/A80 series manufactured by American Kino Industrial Co., Ltd). The Wilhelmy plate method was chosen specifically for the purpose of determining the surface tension of the solution. For each treatment, the process was repeated three times to ensure the accuracy and consistency (Zhao et al., 2022b).

#### 2.2.4 Contact angle

Cotton leaves were affixed to a glass slide using double-sided tape while avoiding leaf veins, lesions, and other imperfections. The contact angle was measured using a drop shape analyzer (DSA100 from KRUSS in Hamburg, Germany). Approximately 2 µL of the pesticide solution was applied to the cotton leaves. Three repetitions were conducted for each treatment to minimize errors throughout the experimental method (Liu et al., 2022).

#### 2.2.5 Spreading rate

Cotton (Huiyuan 720) leaves measured 2 × 2 cm, free from veins and disease spots, and were placed beneath the worktable of a DP74 stereomicroscope (Olympus Co., Ltd., Japan) set at a 10× magnification. Approximately 2 µL of pesticide solution was applied to the leaves, and the spread area of the droplet was measured. The rate of change in the test solution’s spread area was determined based on Equation 3. Three replicates were performed for each treatment.


(3) $$\text{R}=\frac{{\text{S}}_{\text{t}}}{{\text{S}}_{0}}$$

where *S_t_* represents the droplet spread area of *t* (*s*) and *S*_0_ represents the initial area of the droplet.

#### 2.2.6 Toxicity against cotton aphid

The cotton leaves with aphids were collected from an unsprayed cotton field. The collected cotton leaves were dipped into pesticide solutions. After the leaves had been submerged in the solution, the surface water was allowed to evaporate, and the leaves were then placed in Petri plates in an individual manner. To prevent desiccation, damp cotton wool was wrapped around the petiole of each leaf. The Petri plates were maintained at a temperature of 27 ± 1°C with a light–dark cycle of 16:8 h. After 24 h, the dose–mortality response was analyzed and documented.

### 2.3 Droplet deposition characteristics

**Figure 1:**
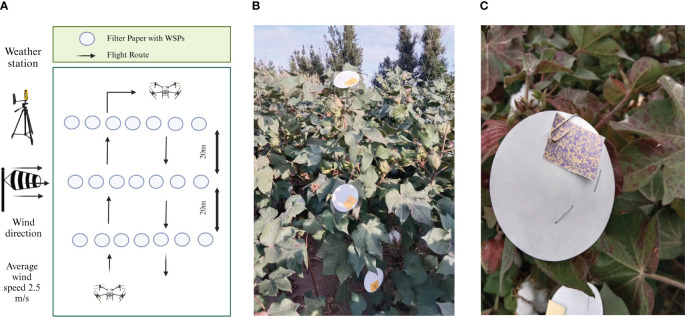
Experimental layout **(A)**, placement of the WSPs at each sampling point **(B)**, and WSP after application of pesticide **(C)**.

The experiment was conducted in a cotton field in Beiquan Town, Shihezi City, Xinjiang, China (44°18′44″ N, 86°3′25″ E). The study area was divided into three sampling lines, each comprising seven sampling points. Each sampling point was divided into three parts: upper, middle, and lower (**Figures 1A, B**). The sampling points were equipped with a water-sensitive paper (WSP) to check the deposition in the cotton field. The WSPs were placed at a distance of 90 cm, 60 cm, and 30 cm from the ground in the upper, middle, and lower layers, respectively. The WSP was chosen because of its effectiveness in providing a visual and quantitative assessment of the droplet distribution and coverage. The WSP was used to evaluate the droplet deposition characteristics such as droplet size, coverage percentage, and droplet density (Wang et al., 2019; Li et al., 2022). A UAV (DJI T50) loaded with a solution of an insecticide and adjuvant was allowed to spray the sampling area. The UAV’s velocity was 5 m/s, and it flew at a height of 3 m above the ground. **Table 3** provides the specific technical specifications of the UAV. Meteorological conditions were assessed with a handheld weather meter, specifically the NK5500. The average wind speed was observed to be 2.5 m/s during the aerial application process while the temperature and relative humidity were 35.1°C and 42.6% respectively.

**Table 3:** Technical parameters of the plant protection UAV used in this study.

Classification	Parameters
Number of rotors	8 (coaxial dual-rotor design)
Rotor diameter	1,375 mm
Spraying load	40 kg
Tank capacity	40 L
Nozzles	LX8060SZ
Spreading flow rate	16 L/min with two nozzles
Effective spray swath	5 m

#### 2.3.1 Processing of water-sensitive papers

The WSPs were collected as soon as the droplets were dried (**Figure 1C**). The collected WSPs were carefully placed in labeled envelopes for further analysis in the laboratory. The WSPs were then scanned using a FileScan2500 scanner (Shanghai Zhongjing Technology Co., Ltd., Shanghai, China) at grayscale and 600 dpi settings. The scanned WSPs were processed using ImageJ 1.38X software from the National Institutes of Health. The analysis included determining droplet size (DV_50_), coverage percentage, and deposition density in the cotton canopy. DV_50_ is commonly used to characterize the spray quality, which represents the volume median diameter and is a critical parameter for evaluating the droplet size distribution. It indicates the droplet size at which half of the spray volume of the droplets are contained in droplets of smaller and larger diameters that help to characterize the spray and understand the size of each classification (Wang et al., 2018b; Zhao et al., 2022a). The data were subsequently compiled and stored for comprehensive analysis.

### 2.4 Droplet drift and average deposition determination

#### 2.4.1 Operational parameters

The experiment was conducted in an empty field at the previously described location. For this experiment, each formulation was tested against the same adjuvant, i.e., nongjianfei, and water as control. Allure red was added to the pesticide solution at the rate of 2% w/v. The DJI T50 UAV was chosen for the test. The experiment involved a set of specific parameters: the spraying volume was fixed at 22.5 L/ha and the UAV was operated at a consistent flying speed of 5 m/s. During the operation, the flight height of the UAV was maintained at 3 m above the ground. The detailed technical parameters of the UAV are given in **Table 3**.

#### 2.4.2 Experimental layout

**Figure 2:**
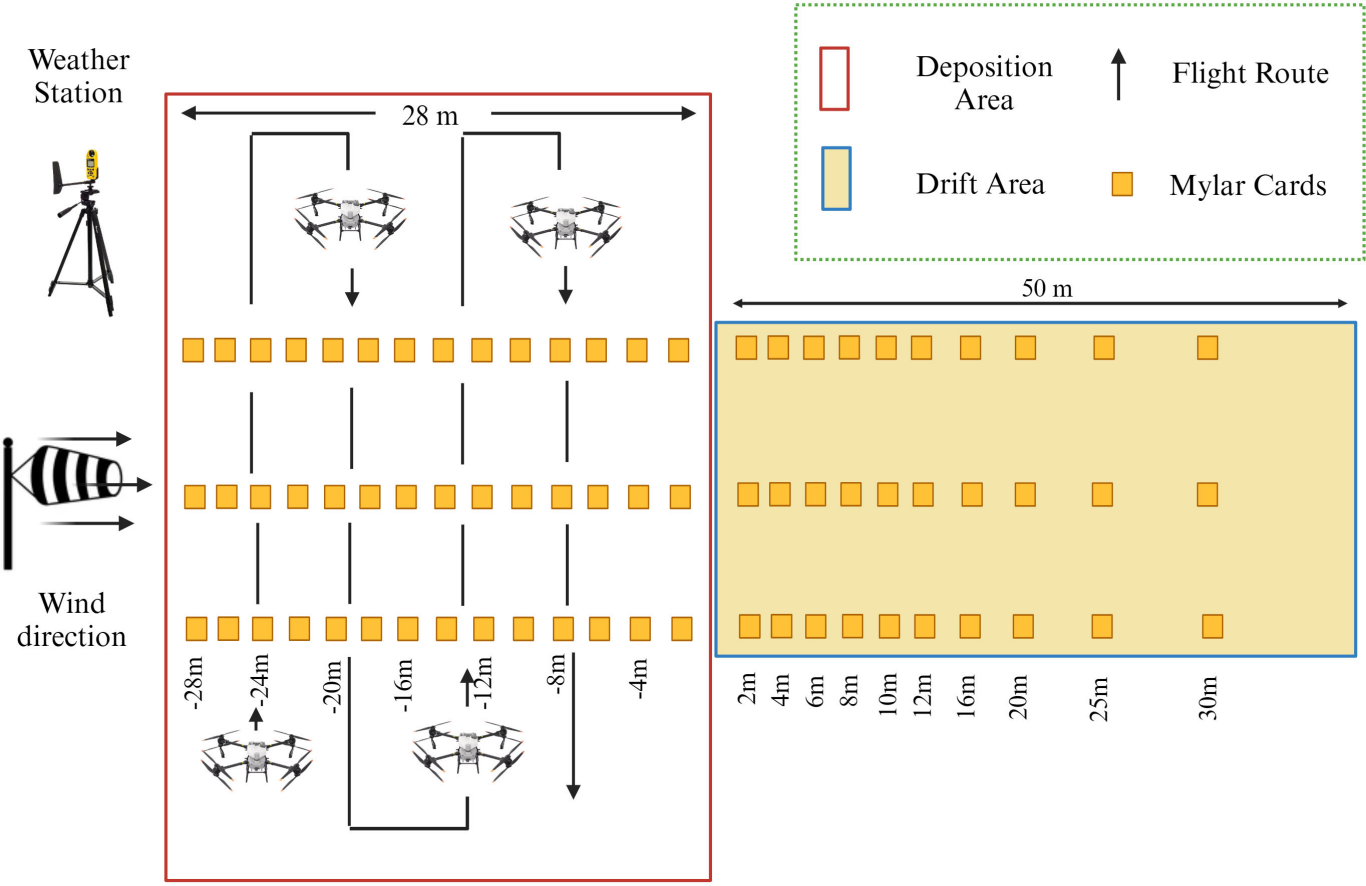
The experimental layout for droplet drift and average deposition determination.

The tested area was divided into two sections, the deposition area and the drift area. Both areas were respectively adopted by mylar card. The mylar cards were arranged parallel to the wind direction. The sampling lines were repeated three times and were spaced 20 m apart from each other. The mylar card in the deposition area collected the droplet deposition and the interval between the mylar cards was 2 m. In the present study the ISO standard 22866:2005 was adopted to suit the specific requirements of the experimental setup. Mylar cards in the drift area were placed 2, 4, 6, 8, 10, 12,16, 20, 25, and 30 m away from the edge of the downwind effective spray width (**Figure 2**). These distances were chosen in order to provide a comprehensive assessment of the droplet drift at several points. The sampling height of the mylar card was set to 1 m above the ground. The height of the UAV from the mylar card was 2 m, and the UAV flew at a height of 3 m from the ground (Wang et al., 2020a). Since the ambient temperature, humidity, wind direction, and wind speed have an important influence on the droplet drift, the NK 5500 (Nielsen-Kellerman in Boothwyn, PA, USA) weather station was used to record the weather conditions. The ambient wind direction was recorded to be consistently moving from west to east throughout the experimental period. During the test, it was ensured that the angle between the wind direction and the working direction was 90 ± 30°. The actual meteorological conditions during the field operation are given in **Table 4**.

**Table 4:** Meteorological conditions for aerial spraying in the field.

Treatments	Pesticide	Adjuvant	Temperature (°C)	Humidity (%)	Wind speed (m/s)
T1	20% acetamiprid SP	Nongjianfei	16.4–17.3	56.6–57.8	0.4–1.0
T2	20% acetamiprid SL	Nongjianfei	14.0–14.9	50.0–51.8	1.9–2.3
T3	10% acetamiprid EC	Nongjianfei	8.8–10.0	45.0–46.4	1.9–2.6
T4	40% acetamiprid WG	Nongjianfei	12.4–12.8	67.1–69.7	1.2–2.1
T5	20% acetamiprid WP	Nongjianfei	13.7–14.6	60.3–61.6	2.6–2.9
T6	5% acetamiprid ME	Nongjianfei	15.3–15.9	57.6–58.9	2.3–3.3
T7	Water	Nongjianfei	16.4–16.9	54.1–55.5	0.9–1.9
T8	Water	–	16.8–17.4	49.2–52.0	2.0–3.0

#### 2.4.3 Sample processing

After the spraying of pesticide solution, the mylar cards, each labeled with specific details regarding the treatment, replication, and site information, were carefully removed and stored in sealed bags. Each sample of the mylar card was washed with 20 mL of distilled water. In order to facilitate the dissolution of the tracer into the water solution, the samples were agitated and vibrated by hand for a duration of 1 min. In previous experiments, it was demonstrated that this process leads to almost complete recovery of the tracer that was put on the materials (Chen et al., 2020b). After elution and vibration, the absorbance value was determined using a full wavelength multifunction microplate reader at a wavelength of 514 nm.

In order to ensure measurement accuracy, six concentrations of allure red 0.2, 0.5, 1.0, 2.0, 5.0, and 10.0 mg/L were formed and set up within the absorbance range. Through the process of linear regression analysis, the equation that describes the linear relationship between the concentration and the absorbance value was derived. The determination coefficient *R* ^2^ was 0.999, which indicated that the concentration of allure red has a good linear relationship with absorbance in the range of determination.

Equation 4 was used to determine the amount of droplet deposition that was deposited at each sampling location after all of the sample concentration values had been measured.


(4) $$\beta_{dep}=\frac{\left(\rho_{smpl}-\rho_{blk}\right)\times F_{cal}\times V_{dil}\;}{\rho_{spray}\;\times \;A_{cal}}$$

where β_dep_ represents the spray deposit or drift (μL/cm^2^), ρ_smpl_ represents the absorbance value, ρ_blk_ represents the absorbance value of the blank control, F_cal_ represents the calibration factor, V_dil_ is the volume of the dilution liquid, A_col_ is the projected area of the collector for catching the spray deposition or drift, and ρ_spray_ is the concentration of the tracer added in spray solution (g/L).

### 2.5 Data analysis

The data were collected using Microsoft Excel 2019 for all tests. One-way analysis of variance (ANOVA) was performed to check the significance of insecticide formulation on density, viscosity, and surface tension. Similarly, one-way ANOVA was done to check the significance of time on contact angle and spreading rate. Means were separated by using the Duncan multiple range test. All the analyses were performed using SPSS 20.0 software.

## 3 Results

### 3.1 Physicochemical properties

#### 3.1.1 Density

**Figure 3:**
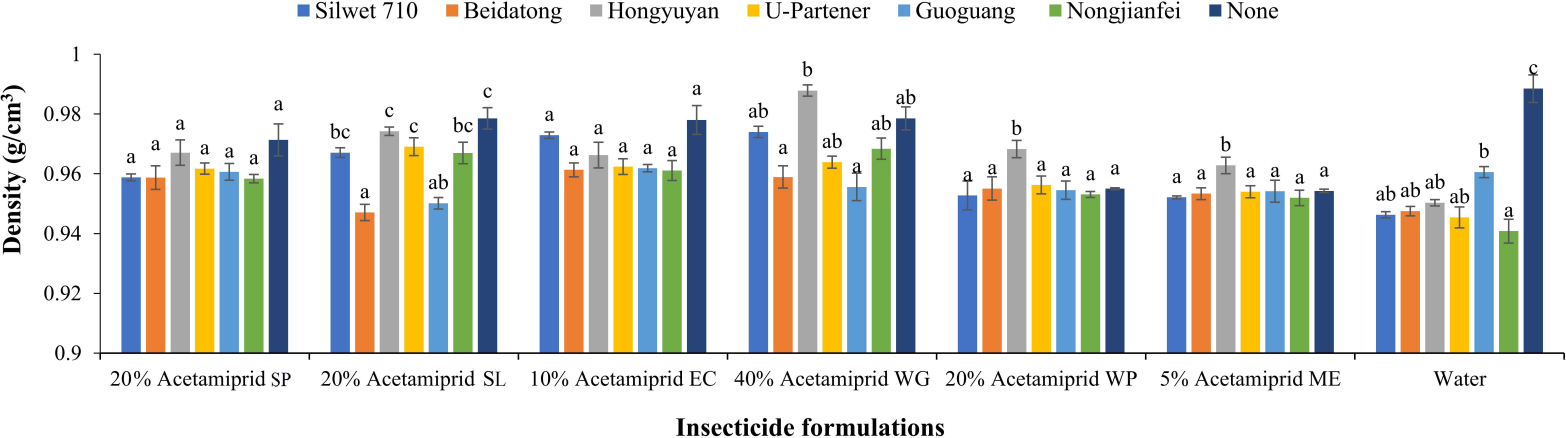
Effects of acetamiprid formulations with adjuvants on solution density. Means sharing similar letters across adjuvants for each formulation are not significantly different at *p* > 0.05.

The pesticide formulations (*F* = 7.22, *p* < 0.05) and adjuvants (*F* = 8.01, *p* < 0.05) showed a significant effect on the density of the solution. However, the interaction of formulations and adjuvants was also significant (*F* = 1.63, *p* < 0.05). The density of all the formulations added with adjuvants is given in **Figure 3**. The results showed that the density of each treatment solution was less than water (0.9885 g/cm^3^). The density of all the treatments ranges from 0.91 to 0.9885 g/cm^3^. A lower density was found when solutions were combined with adjuvants compared to the solution without adjuvant. The 5% acetamiprid ME showed a lower density (0.9542 g/cm^3^) than other formulations. This can reduce its droplet bounce and increase the retention of pesticide solution on the leaves; thus, it can be a potential source for enhancing the droplet deposition characteristics. A higher density was recorded for 40% acetamiprid WG with adjuvants than other formulations. Overall, the adjuvants reduced the densities of all formulations when added with pesticide; hence, they can be a potential source for increasing the droplet deposition. The nongjianfei, when added with 5% acetamiprid ME, gave the least density (0.9519 g/cm^3^) among all combinations of formulation and adjuvants.

#### 3.1.2 Viscosity

**Figure 4:**
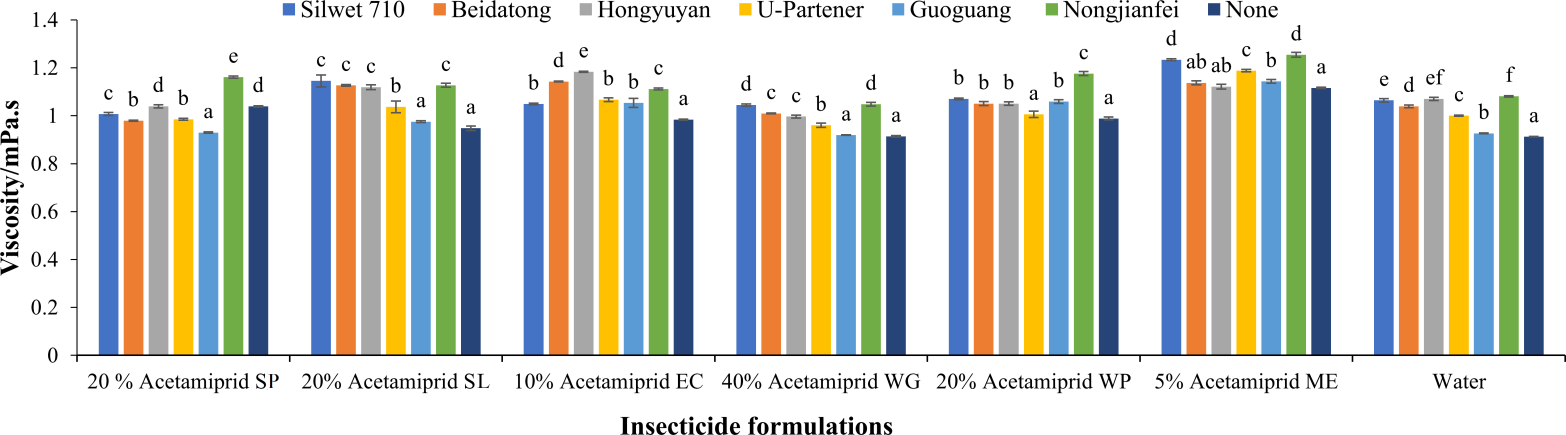
Effects of acetamiprid formulations with adjuvants on solution viscosity. Means sharing similar letters across adjuvants for each formulation are not significantly different at *p* > 0.05.

The pesticide formulations (*F* = 389.08, *p* < 0.05) and adjuvants (*F* = 295.05, *p* < 0.05) showed a significant effect on the viscosity of the liquid. However, the interaction of formulations and adjuvants was also significant (*F* = 28.03, *p* < 0.05). The increase in viscosity decreases the droplet bounce and hence enhances the droplet deposition. **Figure 4** illustrates the viscosity of pesticide formulations and their combination with adjuvants. When the adjuvants were added to pesticide formulations, a great variation in viscosity was observed. The viscosities of formulations, 20% acetamiprid SP, 20% acetamiprid SL, 10% acetamiprid EC, 40% acetamiprid WG, 20% acetamiprid WP, and 5% acetamiprid ME were 1.039 mPa·s, 0.947 mPa·s, 0.984 mPa·s, 0.914 mPa·s, 0.988 mPa·s, and 1.116 mPa·s, respectively, without adjuvants. When these formulations were mixed with adjuvants, the viscosity was enhanced compared to their individual effect. In case of all combinations, the 5% acetamiprid ME showed a higher range of viscosity than other formulations. The best combination was 5% acetamiprid ME with nongjianfei, which increased the viscosity up to 1.254 mPa·s of solution. The viscosity was lower (0.912 mPa·s) using water only, which highlights its limited efficacy in pesticide delivery. The adjuvants increased the viscosity of pesticide formulation and can limit the droplet bounce of the spray solution; hence, they are a potential source of increasing the deposition of pesticide drops on the leaf surface.

#### 3.1.3 Surface tension

**Figure 5:**
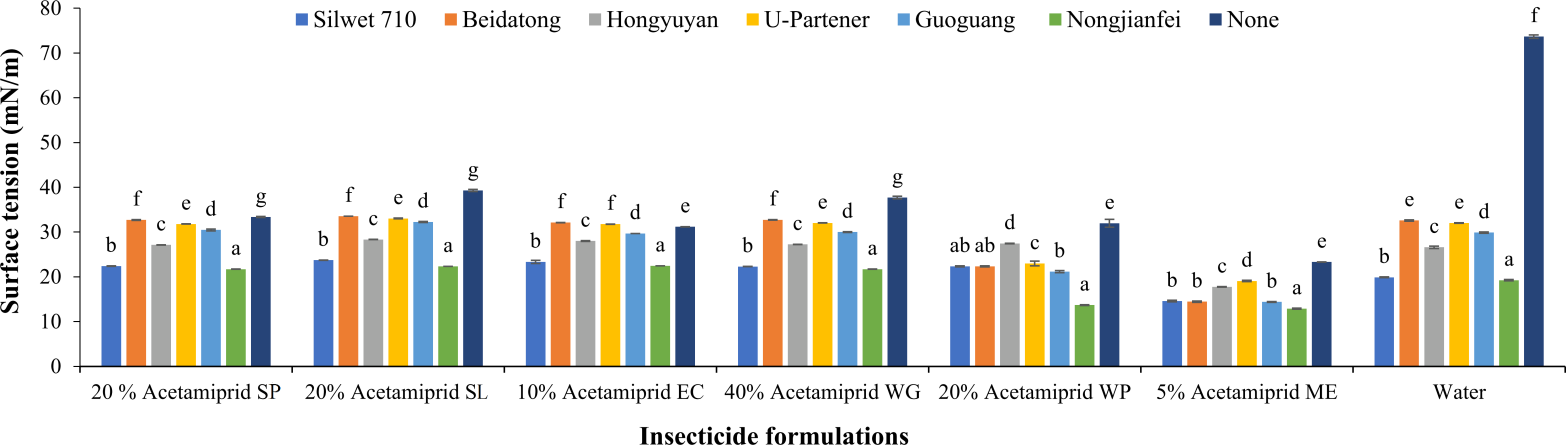
Effects of acetamiprid formulations with adjuvants on surface tension. Means sharing similar letters across adjuvants for each formulation are not significantly different at *p* > 0.05.

The pesticide formulations (*F* = 5,276.48, *p* < 0.05) and adjuvants (*F* = 6,843.65, *p* < 0.05) showed a significant effect on the surface tension. However, the interaction of formulations and adjuvants was also significant (*F* = 905.27, *p* < 0.05). In order to improve the wetting performance and spreading ability of the spraying solution on the leaves, it is possible to reduce the surface tension of the pesticide solution. **Figure 5** shows the surface tension of pesticide formulation, adjuvants, and water as a control. The findings of the present study showed that water had the highest surface tension (73.65 mN/m) among all other treatments. The following trend of surface tension of formulations was recorded: 33.36 mN/m for 20% acetamiprid SP, 39.27 mN/m for 20% acetamiprid SL, 31.17 mN/m for 10% acetamiprid EC, 37.70 mN/m for 40% acetamiprid WG, 31.95 mN/m for 20% acetamiprid WP, and 23.29 mN/m for 5% acetamiprid ME. The 5% acetamiprid ME formulation proved to be very effective in reducing the surface tension. Combining this formulation with the adjuvants decreased the surface tension of the solution compared to other formulations. Notably, the combination of nongjianfei with 5% acetamiprid ME showed the least surface tension (12.88 mN/m) among all combinations. The formulations WG and SC did not perform well in reducing the surface tension when compared with other solutions, yet the adjuvants also reduced their surface tension upon addition.

#### 3.1.4 Contact angle

**Figure 6:**
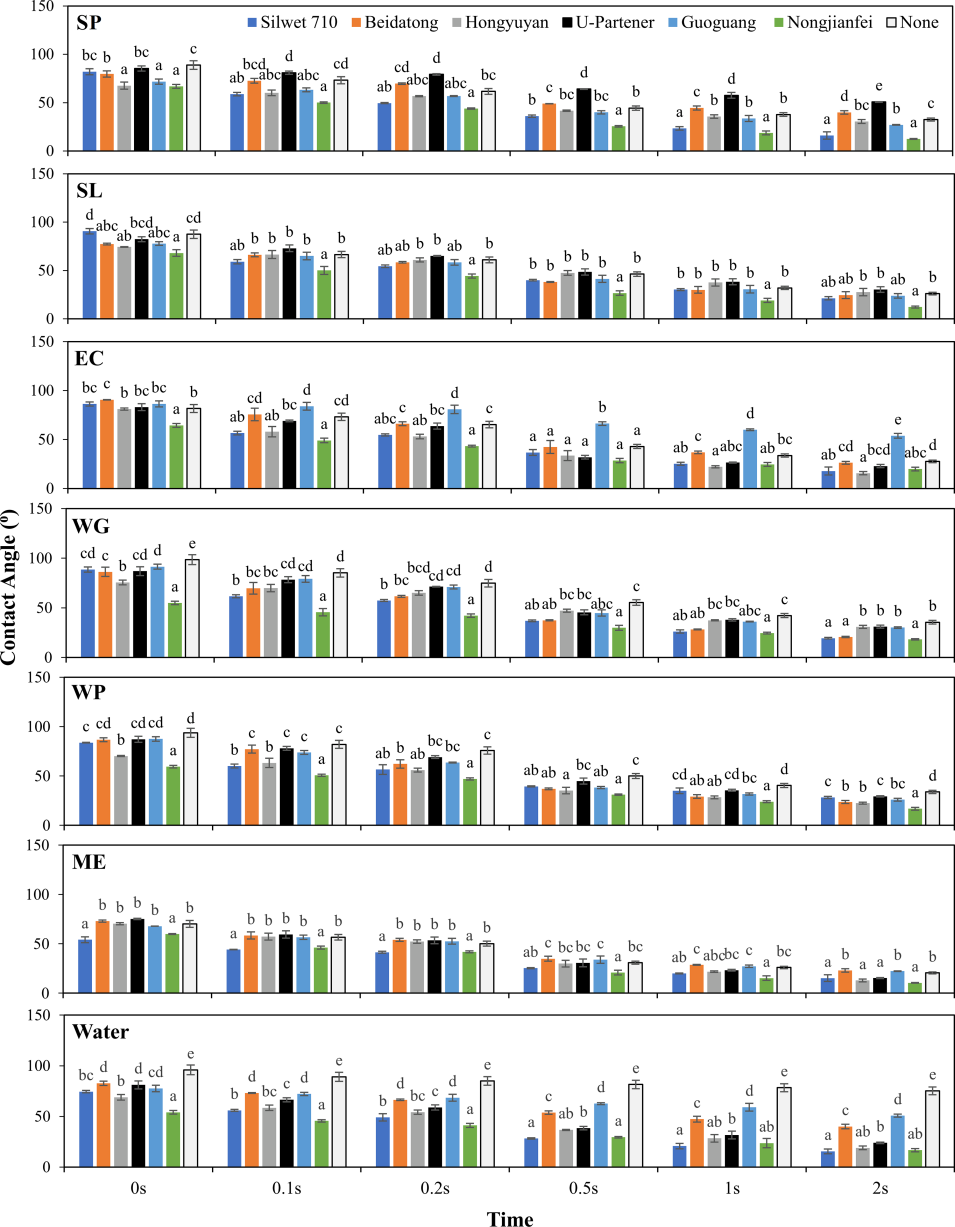
Effects of acetamiprid formulations added with adjuvants on contact angle of the solution. Means sharing similar letters across adjuvants for each formulation are not significantly different at *p* > 0.05.

The detail of the contact angle for the droplets is given in **Figure 6**. The contact angle varies significantly across different formulations and adjuvants and at different time intervals, leading to the fact that these factors play a crucial role in droplet deposition. The contact angle of control treatment (water-only) was substantially higher than the other formulations during all observation times. With the passage of time, the contact angle was decreased. At 2 s, the contact angles for the formulations 20% acetamiprid SP, 20% acetamiprid SL, 10% acetamiprid EC, 40% acetamiprid WG, 20% acetamiprid WP, and 5% acetamiprid ME were 32.52°, 26.18°, 27.65°, 35.44°, 33.79°, and 20.49°, respectively. Acetamiprid 5% ME significantly reduced the contact angle (20.49°) compared to the other formulations without adjuvants. The addition of adjuvants to the pesticide formulation significantly reduced the contact angle. The contact angle of all the combined solutions remains lower than the individual effect of each pesticide formulation. At 2 s, the lower contact angles were observed with 5% acetamiprid ME upon adding different adjuvants compared to the other formulations. The combination of 5% acetamiprid ME with nongjianfei gave the lowest contact angle, 10.39°, suggesting enhanced wettability and spreading, which plays a vital role in effective pesticide application. The results were consistent with a prior study by Meng et al. who demonstrated that the nongjianfei effectively decreases the contact angle of aqueous sprays. Furthermore, the adjuvant notably enhances both droplet coverage and size.

#### 3.1.5 Spreading rate

**Figure 7:**
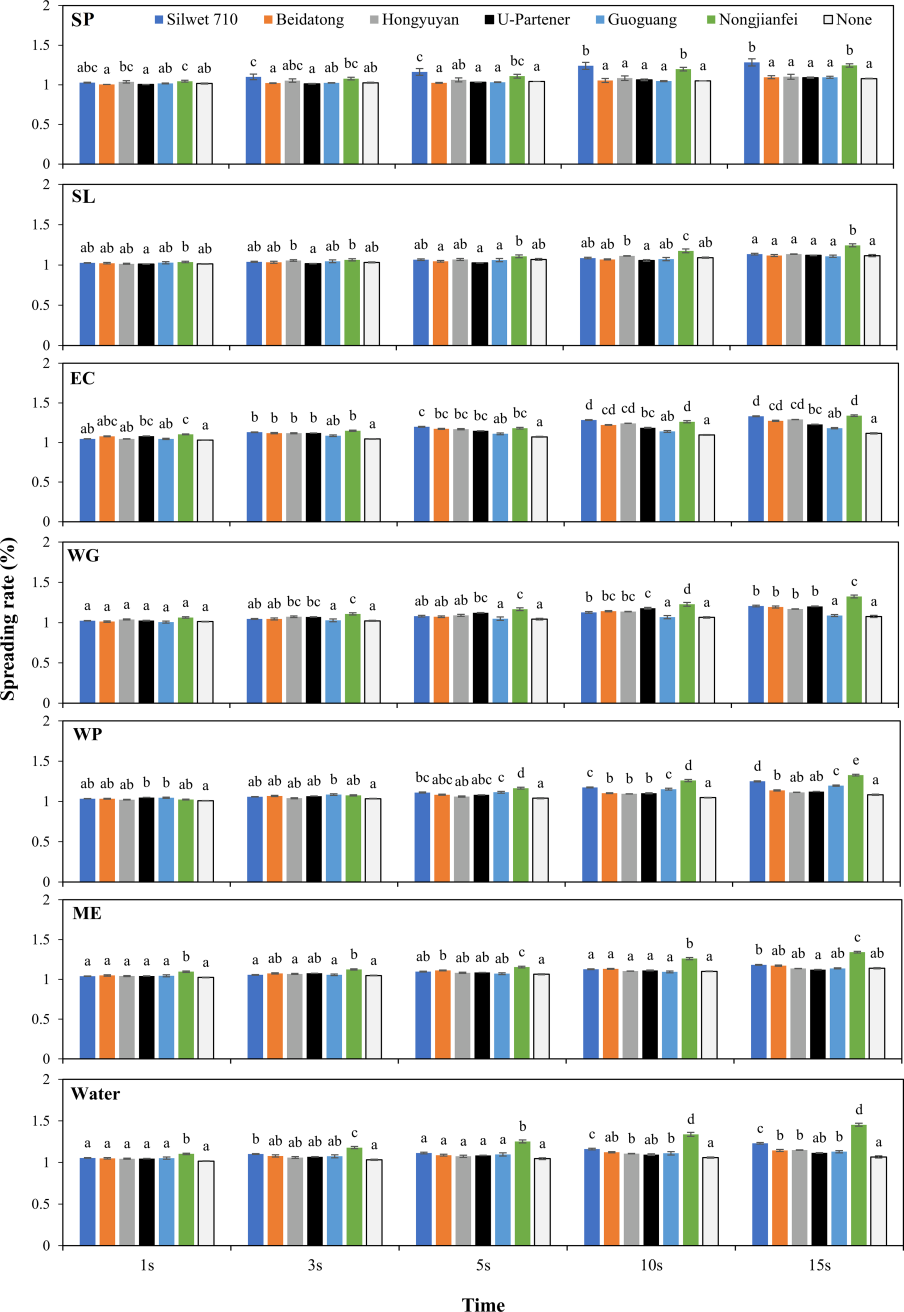
Effects of acetamiprid formulations added with adjuvants on the spreading rate of the solution. Means sharing similar letters across adjuvants for each formulation are not significantly different at *p* > 0.05.

The combination of the leaf surface and the physicochemical features of the pesticide solution is closely related to the wetting and spreading of pesticides on the leaf surface. **Figure 7** illustrates the spreading rate of different solutions containing pesticides and adjuvants over time. At 15 s, the 5% acetamiprid ME showed a higher spreading rate (1.139%), and 40% acetamiprid WG showed the least spreading rate (1.076%). **Figure 7** shows that the choice of an adjuvant can significantly influence the spreading rate by increasing consistently. The pesticide formulations, upon the addition of an adjuvant, increased the spreading rate with the increase in time. The nongjianfei consistently demonstrated a superior performance across different formulations. It can be seen that the ME formulation revealed consistency in spreading the droplet across all adjuvants except U partner, which showed a delayed effect in spreading rate (1.121%). This highlights the positive role of adjuvants in modifying the physicochemical properties of the solution and enhancing the droplet deposition on the leaf surface. Interestingly, water as a control showed the least spreading rate (1.066%) among all other formulations and limits the effect of spreading as well as deposition on the target surface.

#### 3.1.6 Toxicity against cotton aphid

**Figure 8:**
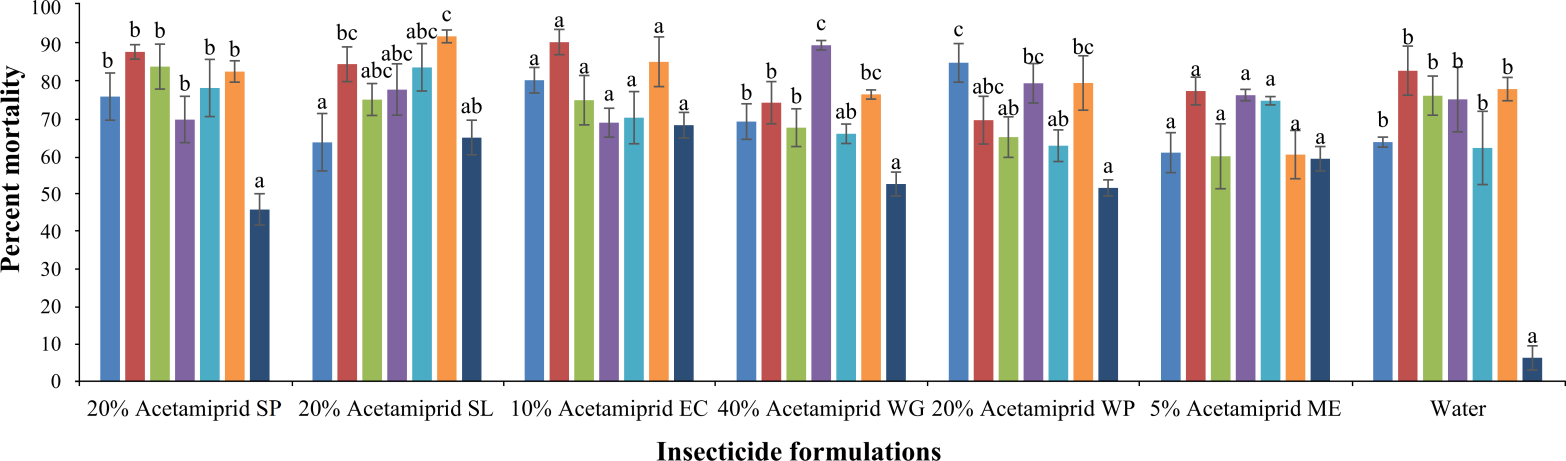
Effects of acetamiprid formulations added with adjuvants on aphid mortality. Means sharing similar letters across adjuvants for each formulation are not significantly different at *p* > 0.05.

The pesticide formulations (*F* = 4.45, *p* < 0.05) and adjuvants (*F* = 17.8, *p* < 0.05) showed a significant effect on the mortality of aphids. However, the interaction of formulations and adjuvants was also significant (*F* = 2.59, *p* < 0.05). The mortality rates of aphids were recorded after the application of several formulations of acetamiprid alone and in combination with adjuvants. The mortality rate was observed after 24 h of pesticide application. The results demonstrated a variability in the efficacy of acetamiprid formulations when they were added with adjuvants (**Figure 8**). The higher mortality (92.27%) was recorded by using 20% SL mixing with nongjianfei. Moreover, 20% acetamiprid SL demonstrated higher efficacy across all adjuvants followed by 10% acetamiprid EC and 5% acetamiprid ME. As shown in **Figure 8**, all the formulations upon mixing with adjuvants showed higher mortality than their individual effect. The least mortality (6.25%) of aphids was recorded in control group.

### 3.2 Droplet deposition characteristics

#### 3.2.1 Droplet size

The size of droplets serves as a critical metric for assessing the quality of spraying operations conducted by UAVs. Improved physicochemical properties can enhance the droplet characteristics. From the laboratory experiments, we found that the addition of adjuvants improved these properties by lowering the surface tension and increasing viscosity, which, in turn, will affect the deposition characteristics. The findings of the study showed that there was a significant effect of pesticides on the droplet size at all three layers, upper (*F* = 16.85, *p* < 0.05), middle (*F* = 22.89, *p* < 0.05), and lower (*F* = 29.68, *p* < 0.05). Adjuvants also significantly affected the droplet size at the upper (*F* = 12.82, *p* < 0.05), middle (*F* = 18.24, *p* < 0.05), and lower (*F* = 19.61, *p* < 0.05) layer. Furthermore, the interaction of pesticide and adjuvants also significantly affected the droplet size (*F* = 2.81, *p* < 0.05 for the upper layer; *F* = 2.32, *p* < 0.05 for the middle layer; and *F* = 1.72, *p* < 0.05 for the lower layer). As shown in **Table 5**, the average droplet size of the treatments with adjuvants was significantly larger than those without adjuvants. The average droplet size of all the formulations, when added with adjuvants, showed varied responses. In the upper layer, using 5% acetamiprid ME, with nongjianfei (270.9 μm) and silwet710 (252.0 μm), led to larger droplets than other formulations. On the other hand, for 20% acetamiprid WP, the lower droplet size (112.0 μm) was found compared to other formulations when no adjuvant was added. In the middle layer, using 5% acetamiprid ME with nongjianfei (263.8 μm) and silwet710 (232.3 μm) showed larger droplets, while beidatong (202.6 μm) and U partner (186.4 μm) produced smaller droplets. In the middle layer, 5% acetamiprid ME alone (without adjuvants) showed a larger droplet size (180.9 μm) when compared with all other formulations. Here, 20% acetamiprid WP also gave the lowest droplet size (109.0 μm) with water found. In the lower layer, using 5% acetamiprid ME, the highest droplet size was recorded with nongjianfei (253.7 μm) and silwet710 (222.4 μm). When compared with other formulations using no adjuvant, 5% acetamiprid ME gave a higher droplet size (171.0 μm), while 20% acetamiprid WP remained the least performer with a droplet size of 98.0 μm. Overall, 5% acetamiprid ME performed better with all adjuvants by showing a larger droplet size in all layers and, hence, proved to be a good formulation in enhancing the droplet deposition and reducing drift because of its increased droplet size.

**Table 5:** Effect of acetamiprid formulation added with adjuvants on the droplet size (μm) sprayed by UAV.

Droplet	Pesticides	Silwet 710	Beidatong	Hongyuyan	U Partener	Guoguang	Nongjianfei	None	Sign.
Upper	20% acetamiprid SP	227.2 ± 17.2ab	204.1 ± 13.4a	202.7 ± 10.3a	225.0 ± 11.1ab	204.1 ± 25.1a	252.4 ± 9.8b	188.1 ± 11.9a	*F* = 2.10**
	20% acetamiprid SL	210.0 ± 12.5b	169.3 ± 14.7ab	213.4 ± 28.9b	188.5 ± 12.8b	206.5 ± 22.4b	218.0 ± 18.6b	128.4 ± 9.8a	*F* = 3.12**
	10% acetamiprid EC	213.5 ± 6.4ab	232.4 ± 29.2ab	182.5 ± 16.1a	181.9 ± 13.9a	202.5 ± 6.4ab	241.1 ± 17.4b	189.0 ± 19.9ab	*F* = 1.91**
	40% acetamiprid WG	210.2 ± 16.4a	200.3 ± 44.1a	165.0 ± 9.8a	155.0 ± 11.5a	208.1 ± 44.9a	225.7 ± 17.2b	146.3 ± 11.8a	*F* = 1.38^ns^
	20% acetamiprid WP	165.1 ± 20.8c	145.0 ± 12.6abc	160.0 ± 18.5bc	120.5 ± 8.0ab	151.0 ± 12.8abc	216.5 ± 12.9d	112.0 ± 9.1a	*F* = 5.76***
	5% acetamiprid ME	252.0 ± 11.6ab	229.3 ± 34.1ab	237.0 ± 24.2ab	224.6 ± 23.0ab	201.7 ± 14.3a	270.9 ± 14.3b	196.0 ± 13.0a	*F* = 1.63^ns^
	Water	483.8 ± 109.1c	208.6 ± 15.0ab	203.8 ± 11.1ab	247.4 ± 12.3ab	331.9 ± 83.7bc	513.6 ± 97.0c	125.0 ± 9.7a	*F* = 5.30***
Middle	20% acetamiprid SP	220.7 ± 13.7b	197.1 ± 13.6ab	195.2 ± 11.1ab	223.9 ± 6.7b	189.3 ± 17.4ab	227.2 ± 14.9b	168.5 ± 12.4a	*F* = 2.68**
	20% acetamiprid SL	182.0 ± 14.1b	165.2 ± 13.1b	181.1 ± 15.5b	186.7 ± 16.8b	196.0 ± 14.1b	205.7 ± 12.8b	123.8 ± 9.0a	*F* = 3.72**
	10% acetamiprid EC	208.8 ± 7.1ab	228.1 ± 25.3b	172.4 ± 14.1a	169.8 ± 14.5ab	179.3 ± 7.2ab	224.6 ± 19.3b	177.9 ± 17.9a	*F* = 2.41**
	40% acetamiprid WG	182.1 ± 12.1ab	177.7 ± 17.4ab	162.5 ± 14.0a	144.6 ± 14.2a	146.4 ± 11.1a	213.4 ± 19.3b	137.9 ± 14.3a	*F* = 3.21**
	20% acetamiprid WP	143.9 ± 17.0b	131.8 ± 19.6a	147.7 ± 14.8a	116.0 ± 9.8ab	145.7 ± 19.8ab	188.2 ± 9.5b	109.0 ± 10.2a	*F* = 2.96**
	5% acetamiprid ME	232.3 ± 13.6bc	202.6 ± 16.1ab	218.0 ± 17.1ab	186.4 ± 13.5a	211.1 ± 12.9ab	263.8 ± 11.7c	180.9 ± 14.7a	*F* = 3.88**
	Water	329.9 ± 86.1cd	222.5 ± 13.8b	246.9 ± 9.6bc	244.9 ± 9.1bc	195.3 ± 12.3ab	370.3 ± 20.0d	108.0 ± 9.6a	*F* = 6.17***
Lower	20% acetamiprid SP	170.0 ± 12.2ab	193.2 ± 12.1abc	177.6 ± 13.4ab	202.1 ± 11.4bc	172.2 ± 11.5ab	219.3 ± 8.0c	163.7 ± 13.6a	*F* = 2.86**
	20% acetamiprid SL	151.5 ± 12.9abc	133.2 ± 15.0ab	167.1 ± 18.2bc	186.2 ± 15.8c	167.9 ± 14.2bc	192.3 ± 14.6c	120.3 ± 6.1a	*F* = 3.41**
	10% acetamiprid EC	166.7 ± 10.9ab	194.7 ± 13.1ab	165.7 ± 15.4ab	151.9 ± 13.4a	156.0 ± 9.5a	202.6 ± 20.9b	162.9 ± 15.5ab	*F* = 1.78^ns^
	40% acetamiprid WG	175.9 ± 14.8bc	126.6 ± 9.0a	153.7 ± 13.1ab	150.1 ± 17.6ab	144.4 ± 12.2ab	202.6 ± 16.6c	126.5 ± 12.8a	*F* = 3.77**
	20% acetamiprid WP	131.8 ± 13.9a	122.1 ± 20.2a	126.1 ± 9.51a	110.9 ± 10.6a	110.4 ± 8.41a	171.7 ± 10.3b	98.00 ± 4.80a	*F* = 3.8**
	5% acetamiprid ME	222.4 ± 13.6bc	189.2 ± 13.9ab	209.0 ± 12.1ab	193.8 ± 11.4ab	204.7 ± 14.5ab	253.7 ± 12.3c	171.0 ± 10.7a	*F* = 4.31**
	Water	196.9 ± 13.4b	209.1 ± 11.0b	215.3 ± 14.6b	201.6 ± 15.5b	200.5 ± 13.5b	240.6 ± 20.2b	101.0 ± 8.9a	*F* = 9.43***

** shows the significance at p < 0.05, *** shows the significance at p < 0.01, ns shows the non-significance. Means sharing similar letters in a row are not significant at p > 0.05. Means sharing similar letters across adjuvants for each formulation (in a row) are not significantly different at p > 0.05.

#### 3.2.2 Droplet coverage

The coverage percentage of acetamiprid formulation combined with various adjuvants exhibited significant variations across the upper, middle, and lower layers. Pesticides showed a significant impact on coverage percentage in the upper (*F* = 23.97, *p* < 0.05), middle (*F* = 8.59, *p* < 0.05), and lower (*F* = 10.86, *p* < 0.05) layers. Similarly, adjuvants significantly influenced coverage percentage in the upper (*F* = 23.99, *p* < 0.05), middle (*F* = 20.88, *p* < 0.05), and lower (*F* = 19.83, *p* < 0.05) layers. Moreover, the interaction between pesticides and adjuvants played a significant role in coverage percentage (*F* = 5.25, *p* < 0.05 for the upper layer; *F* = 3.65, *p* < 0.05 for the middle layer; and *F* = 4.62, *p* < 0.05 for the lower layer). In the upper layer, 5% acetamiprid ME with nongjianfei resulted in a significantly higher coverage (10.6%), while guoguang showed a lower coverage (5.19%). Using 5% acetamiprid ME without any adjuvant gave a higher coverage than all other formulations of acetamiprid. With 20% acetamiprid SP, a lower coverage (2.35%) was observed in the upper layer without adjuvants. Notably, the none category (without adjuvants) acetamiprid consistently displayed a lower coverage compared to adjuvants across all formulations. In the middle layer, nongjianfei demonstrated a higher coverage (6.17%) for 5% acetamiprid ME, while guoguang exhibited a lower coverage (4.04%). Here, 5% acetamiprid ME still exhibited a higher coverage (3.91%) after comparing it with all other formulations when no adjuvant was added. At the same time, the performance of 5% acetamiprid ME with all adjuvants was better than other formulations. Among all formulations, 40% acetamiprid WG showed a lower coverage followed by the 20% acetamiprid WP. In the lower layer, a similar trend was observed: 5% acetamiprid ME performed better with all adjuvants. On the other hand, water as a control remained the least performer among all treatments. The presence or absence and the type of adjuvant caused a significant impact on droplet coverage. The results showed that the none category (no adjuvant) caused a lower coverage compared to adjuvants across all three layers (**Table 6**).

**Table 6:** Effect of acetamiprid formulation added with adjuvants on the coverage (%) sprayed by UAV.

Droplet	Pesticides	Silwet 710	Beidatong	Hongyuyan	U Partener	Guoguang	Nongjianfei	None	Sign.
Upper	20% acetamiprid SP	5.2 ± 1.0b	3.4 ± 0.7ab	3.6 ± 0.6ab	2.4 ± 0.3a	4.4 ± 1.3ab	5.3 ± 0.6b	2.3 ± 0.4a	*F* = 2.46**
	20% acetamiprid SL	6.4 ± 1.0ab	9.3 ± 1.8bc	3.9 ± 1.0a	3.4 ± 0.6a	8.1 ± 0.9bc	9.9 ± 0.4c	4.6 ± 1.0a	*F* = 5.78***
	10% acetamiprid EC	5.5 ± 0.5b	3.9 ± 0.6ab	2.5 ± 0.2a	5.1 ± 1.3b	2.6 ± 0.1a	8.2 ± 0.4c	2.5 ± 0.3a	*F* = 10.1***
	40% acetamiprid WG	7.2 ± 1.4bc	8.5 ± 2.2c	3.4 ± 1.1ab	4.5 ± 1.0ab	9.3 ± 1.1c	9.8 ± 0.6c	3.0 ± 0.7a	*F* = 4.74***
	20% acetamiprid WP	6.6 ± 1.3b	2.8 ± 0.5a	7.3 ± 0.7b	7.8 ± 1.4b	8.6 ± 1.9b	8.9 ± 0.5b	2.4 ± 0.4a	*F* = 5.43***
	5% acetamiprid ME	9.3 ± 0.7b	7.6 ± 1.9ab	7.0 ± 1.5ab	9.9 ± 1.5b	5.1 ± 1.0a	10. ± 0.7b	5.0 ± 0.6a	*F* = 3.13**
	water	24.4 ± 4.6c	10.4 ± 2.0ab	4.6 ± 0.9a	4.1 ± 1.2a	14.9 ± 3.2b	23.2 ± 3.9c	2.3 ± 0.3a	*F* = 10.7***
Middle	20% acetamiprid SP	2.8 ± 0.5abc	1.7 ± 0.3a	2.2 ± 0.4abc	1.9 ± 0.5ab	3.4 ± 0.7bc	3.6 ± 0.5c	2.4 ± 0.5abc	*F* = 1.92**
	20% acetamiprid SL	5.3 ± 0.2bc	5.6 ± 1.2bc	1.5 ± 0.4a	1.9 ± 0.4a	7.7 ± 1.5cd	8.3 ± 0.7d	3.2 ± 0.4ab	*F* = 9.61***
	10% acetamiprid EC	3.4 ± 0.3ab	3.1 ± 1.0ab	2.4 ± 0.7ab	4.1 ± 0.6b	2.5 ± 0.3ab	6.4 ± 0.5c	1.9 ± 0.2a	*F* = 5.81***
	40% acetamiprid WG	3.8 ± 0.6ab	6.6 ± 1.9c	1.7 ± 0.3a	2.7 ± 0.7ab	5.4 ± 0.8bc	6.7 ± 0.7c	1.2 ± 0.2a	*F* = 5.64***
	20% acetamiprid WP	4.5 ± 1.3ab	1.8 ± 0.3a	4.4 ± 1.6ab	4.7 ± 1.1abc	8.6 ± 2.4c	7.7 ± 0.4bc	2.1 ± 0.4a	*F* = 3.66**
	5% acetamiprid ME	5.3 ± 1.0a	5.0 ± 0.7a	4.3 ± 0.9a	5.3 ± 0.7a	4.0 ± 0.6a	6.1 ± 0.5a	3.9 ± 0.6a	*F* = 1.12ns
	water	12.9 ± 3.5b	4.9 ± 1.2a	1.8 ± 0.2a	2.8 ± 1.0a	5.6 ± 0.8a	12.9 ± 2.0b	1.9 ± 0.2a	*F* = 8.38***
Lower	20% acetamiprid SP	1.9 ± 0.2ab	1.1 ± 0.1a	1.2 ± 0.0a	1.3 ± 0.0a	2.5 ± 0.5b	2.5 ± 0.3b	1.7 ± 0.3ab	*F* = 3.68**
	20% acetamiprid SL	4.5 ± 0.4bc	4.2 ± 0.9bc	1.0 ± 0.1a	1.5 ± 0.3a	5.0 ± 1.2bc	5.6 ± 0.7c	2.9 ± 0.6ab	*F* = 5.72***
	10% acetamiprid EC	1.4 ± 0.2a	1.4 ± 0.3a	1.1 ± 0.1a	3.9 ± 1.2b	1.1 ± 0.1a	4.3 ± 0.4b	1.2 ± 0.1a	*F* = 6.91***
	40% acetamiprid WG	2.5 ± 0.5abc	4.2 ± 1.0bc	1.3 ± 0.2a	1.7 ± 0.4a	3.1 ± 0.7abc	4.5 ± 0.6c	2.2 ± 0.7ab	*F* = 3.21**
	20% acetamiprid WP	1.9 ± 0.4ab	1.2 ± 0.3a	1.8 ± 0.2ab	3.4 ± 0.9bc	3.2 ± 0.7bc	4.2 ± 0.6c	1.3 ± 0.3a	*F* = 4.01**
	5% acetamiprid ME	2.8 ± 0.8ab	2.2 ± 0.4a	3.7 ± 1.0ab	4.6 ± 0.8b	2.0 ± 0.3a	4.5 ± 0.6b	3.2 ± 0.4ab	*F* = 2.14**
	water	8.0 ± 1.5c	2.8 ± 0.6ab	1.3 ± 0.1a	1.2 ± 0.1a	4.6 ± 1.1b	9.5 ± 1.4c	1.1 ± 0.1a	*F* = 13.1***

** shows the significance at p < 0.05, *** shows the significance at p < 0.01, ns shows the non-significance. Means sharing similar letters in a row are not significant at p > 0.05. Means sharing similar letters across adjuvants for each formulation (in a row) are not significantly different at p > 0.05.

#### 3.2.3 Droplet density

The droplet density of the formulations varies among the layers of the cotton canopy. The findings showed that pesticides had a significant impact on the droplet density in the upper (*F* = 9.07, *p* < 0.05), middle (*F* = 9.19, *p* < 0.05), and lower (*F* = 15.3, *p* < 0.05) layers. Similarly, the adjuvants had a significant effect on the droplet density in the upper (*F* = 16.26, *p* < 0.05), middle (*F* = 13.5, *p* < 0.05), and lower (*F* = 12.8, *p* < 0.05) layers. The interaction between pesticides and adjuvants contributed significantly to density variations (*F* = 1.58, *p* < 0.05 for the upper layer; *F* = 1.84, *p* < 0.05 for the middle layer; and *F* = 2.41, *p* < 0.05 for the lower layer). In the upper layer, the density was higher than in the middle and lower layers. The density of the solutions added with adjuvants was greater than the individual effect of each formulation. In the upper layer, 5% acetamiprid ME gave a higher density (256.6 deposits/cm^2^) when compared with other formulations. The combined effect of 5% acetamiprid ME with nongjianfei was better than the individual effect in terms of droplet density (429.9 deposits/cm^2^). This formulation showed a higher density with all adjuvants in the upper layer. Notably, the 20% acetamiprid SP and 10% acetamiprid EC showed lower densities, 137.6 deposits/cm^2^ and 146.9 deposits/cm^2^ respectively. Their combined effect was also lower than the other formulations. In the middle and lower layers, the results varied significantly, as 5% acetamiprid ME showed a higher density while 20% acetamiprid SP and 40% acetamiprid WG showed lower densities. Their densities in the middle and lower layers were 129.9 deposits/cm^2^ and 94.65 deposits/cm^2^, and 70.62 deposits/cm^2^ and 90.10 deposits/cm^2^, respectively. Compared to other formulations, their combined effects were also lower in these layers (**Table 7**). Overall, nongjianfei demonstrated a higher droplet density across various formulations and layers, indicating its efficacy in enhancing droplet coverage. Moreover, the none category exhibited a lower droplet density compared to the combination of pesticides and adjuvants, underscoring the importance of adjuvants in optimizing droplet distribution.

**Table 7:** Effect of acetamiprid formulation added with adjuvants on the droplet density (deposits/cm^2^) sprayed by UAV.

Droplet	Pesticides	Silwet 710	Beidatong	Hongyuyan	U Partener	Guoguang	Nongjianfei	None	Sign.
Upper	20% acetamiprid SP	283.9 ± 49.5b	212.2 ± 49.8ab	233.4 ± 49.1ab	145.4 ± 26.9a	225.8 ± 45.1ab	318.1 ± 31.1b	137.6 ± 33.6a	2.51**
	20% acetamiprid SL	328.5 ± 36.0abc	368.1 ± 47.9bc	242.5 ± 48.8ab	231.5 ± 43.4a	379.0 ± 48.5c	390.6 ± 34.1c	227.7 ± 37.3a	2.96**
	10% acetamiprid EC	253.4 ± 35.1ab	189.2 ± 36.9ab	183.2 ± 27.5ab	280.8 ± 52.1b	199.8 ± 14.7ab	294.0 ± 38.1b	146.9 ± 34.7a	2.38**
	40% acetamiprid WG	353.6 ± 59.7bcd	314.8 ± 45.8abc	206.7 ± 51.1ab	307.6 ± 62.1abc	452.0 ± 38.1cd	470.0 ± 52.1d	190.6 ± 34.9a	4.68***
	20% acetamiprid WP	390.8 ± 59.3bc	247.8 ± 48.6ab	344.7 ± 50.9abc	373.7 ± 40.6bc	277.1 ± 47.8abc	402.1 ± 37.2c	225.2 ± 38.4a	2.38**
	5% acetamiprid ME	369.9 ± 35.2a	288.4 ± 52.7a	251.7 ± 47.6a	394.3 ± 40.3a	270.3 ± 43.4a	429.9 ± 22.3a	256.6 ± 26.3a	1.17ns
	water	396.2 ± 42.1c	384.0 ± 44.6c	257.9 ± 38.8bc	234.3 ± 30.3ab	350.8 ± 49.1cd	417.0 ± 18.3c	140.2 ± 13.9a	8.07***
Middle	20% acetamiprid SP	165.3 ± 33.2ab	130.0 ± 20.4ab	137.2 ± 26.8ab	71.6 ± 34.2a	154.7 ± 34.1ab	179.3 ± 28.7b	129.9 ± 34.1ab	1.29ns
	20% acetamiprid SL	191.9 ± 23.1ab	205.4 ± 28.2b	168.7 ± 28.6ab	100.7 ± 28.9a	316.4 ± 48.6c	251.1 ± 35.2bc	167.2 ± 21.9ab	4.63***
	10% acetamiprid EC	167.8 ± 16.3a	119.6 ± 42.2a	129.0 ± 42.7a	213.8 ± 41.9a	172.3 ± 21.9a	220.7 ± 36.7a	131.1 ± 30.1a	1.40ns
	40% acetamiprid WG	224.2 ± 43.8bc	251.7 ± 47.2cd	121.3 ± 26.1ab	190.4 ± 48.1abc	340.8 ± 34.0d	347.2 ± 44.1d	94.6 ± 22.0a	6.30***
	20% acetamiprid WP	251.9 ± 48.5a	192.4 ± 21.8a	256.3 ± 48.9a	249.5 ± 41.1a	227.4 ± 37.8a	297.9 ± 27.8a	185.4 ± 41.0a	0.99ns
	5% acetamiprid ME	294.3 ± 42.7b	269.9 ± 45.6ab	159.1 ± 37.8a	302.3 ± 46.4b	253.9 ± 39.5ab	307.3 ± 27.4b	164.4 ± 21.9a	2.71**
	water	333.5 ± 31.5c	206.2 ± 42.2ab	144.5 ± 15.8a	179.1 ± 42.2a	287.4 ± 46.8bc	349.1 ± 37.3c	108.3 ± 26.6a	6.84***
Lower	20% acetamiprid SP	100.6 ± 24.4ab	77.6 ± 14.4ab	73.22 ± 2.4ab	36.8 ± 8.7a	129.6 ± 29.7b	131.1 ± 32.7b	70.6 ± 19.3ab	2.53**
	20% acetamiprid SL	132.1 ± 19.0abc	171.2 ± 26.4bcd	108.6 ± 7.6ab	72.7 ± 27.4a	235.1 ± 50.2d	192.5 ± 15.1cd	103.6 ± 14.8ab	4.68***
	10% acetamiprid EC	84.3 ± 19.8ab	63.0 ± 30.7a	46.28 ± 10.8a	120.9 ± 20.5b	57.7 ± 9.4a	91.58 ± 21.2ab	49.2 ± 10.3a	2.03**
	40% acetamiprid WG	151.6 ± 38.3abc	214.7 ± 44.5bc	87.15 ± 27.5a	104.6 ± 34.4ab	232.2 ± 59.1c	209.7 ± 17.3bc	90.1 ± 20.7a	2.89**
	20% acetamiprid WP	152.1 ± 33.1abc	108.3 ± 17.3a	119.3 ± 10.2ab	209.4 ± 44.7bc	202.6 ± 41.0abc	220.2 ± 38.7c	106.1 ± 20.8a	2.48**
	5% acetamiprid ME	178.8 ± 30.1a	157.1 ± 26.6a	126.8 ± 34.6a	188.6 ± 28.2a	122.5 ± 31.5a	199.5 ± 17.7a	131.3 ± 32.7a	1.17ns
	water	318.1 ± 38.5c	145.9 ± 32.9ab	126.1 ± 9.4ab	134.1 ± 8.81ab	203.9 ± 45.4b	321.5 ± 31.9c	79.9 ± 11.3a	10.8***

** shows the significance at p < 0.05, *** shows the significance at p < 0.01, ns shows the non-significance. Means sharing similar letters in a row are not significant at p > 0.05. Means sharing similar letters across adjuvants for each formulation (in a row) are not significantly different at p > 0.05.

### 3.3 Droplet drift and average deposition

#### 3.3.1 Droplet drift

**Figure 9:**
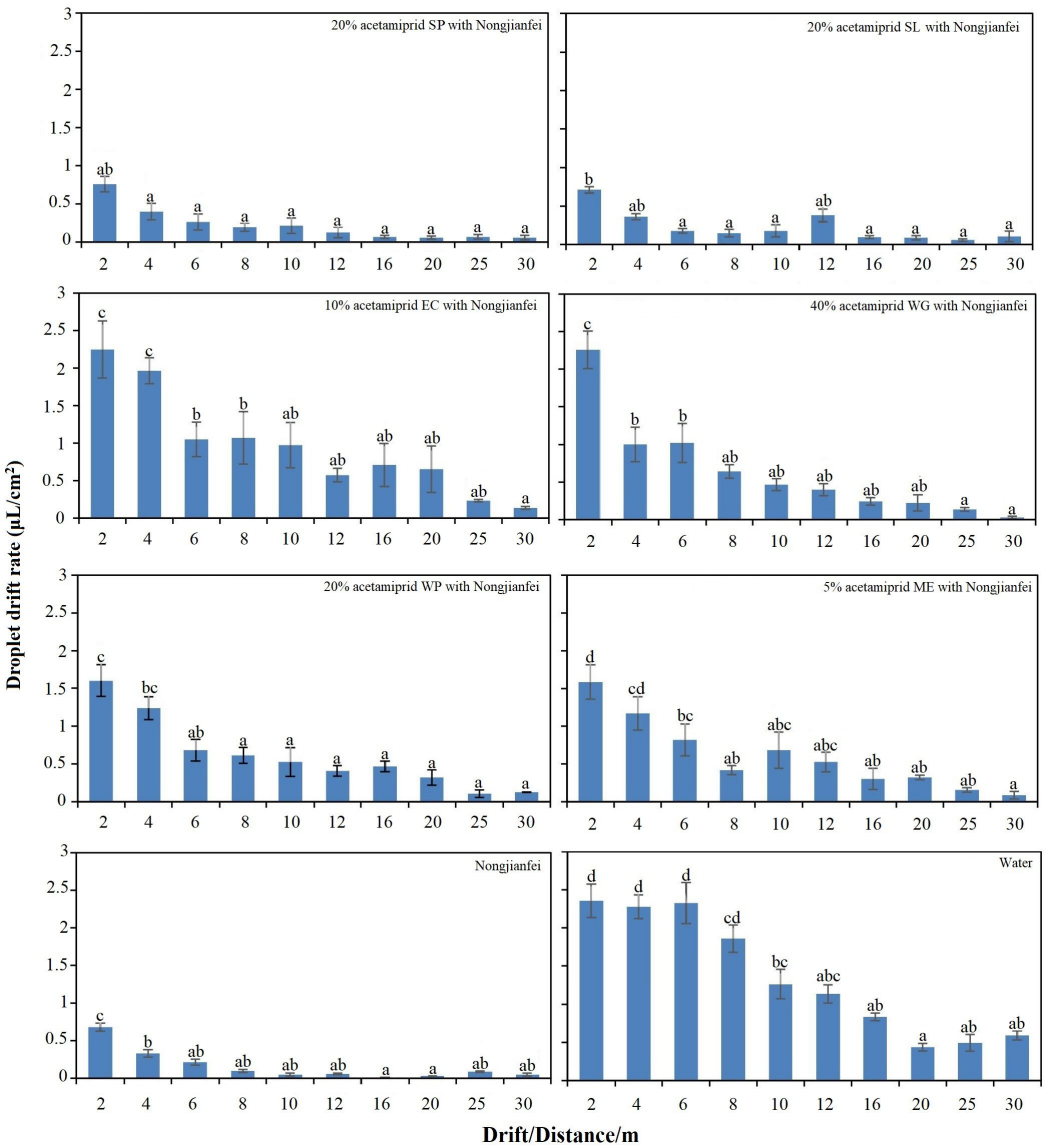
Droplet drift for all treatments in the non-target area. Means sharing similar letters are not significantly different at *p* > 0.05.

The atomization process of the pesticide liquid is complex and it is significantly influenced by adding adjuvants. The adjuvants alter the physicochemical properties such as viscosity, surface tension, and contact angle, thereby impacting the process of atomization. The findings of previous experiments corroborate these effects, demonstrating that these modifications such as increased viscosity, reduced surface tension, and lowered contact angle led to a lower drift of the pesticide droplets. The treatments (*F* = 43.84, *p* < 0.05) and distances (*F* = 40.19, *p* < 0.05) showed a significant effect on the droplet drift. The distribution of droplet drift with different formulations of acetamiprid added with nongjianfei at different distances within the drift area is given in **Figure 9**. All the formulations showed different drift rates at different wind speeds (**Table 4**). When the wind speed was lower, the drift rate was relatively low (**Figure 9**). At the same time, when the wind speed was higher, the droplets showed a higher drift. At a higher wind speed (2.3–3.3 m/s), the formulation 5% acetamiprid ME with nongjianfei showed the least drift (1.586 μL/cm^2^) among all other treatments at 2 m within the drift area. At a wind speed of 1.2–2.1 m/s, 40% acetamiprid WG gave the highest droplet drift (2.257 μL/cm^2^) among all other formulations of acetamiprid. The nongjianfei alone showed the lowest drift (0.681 μL/cm^2^) among all the treatments at 2 m. Similarly, the drift rate at all other points in the drift area for nongjianfei was lower. The highest drift (2.355 μL/cm^2^) was observed at 2 m for water alone, and it also gave the highest drift for all distances within the drift area (**Figure 9**).

#### 3.3.2 Average droplet deposition

**Figure 10:**
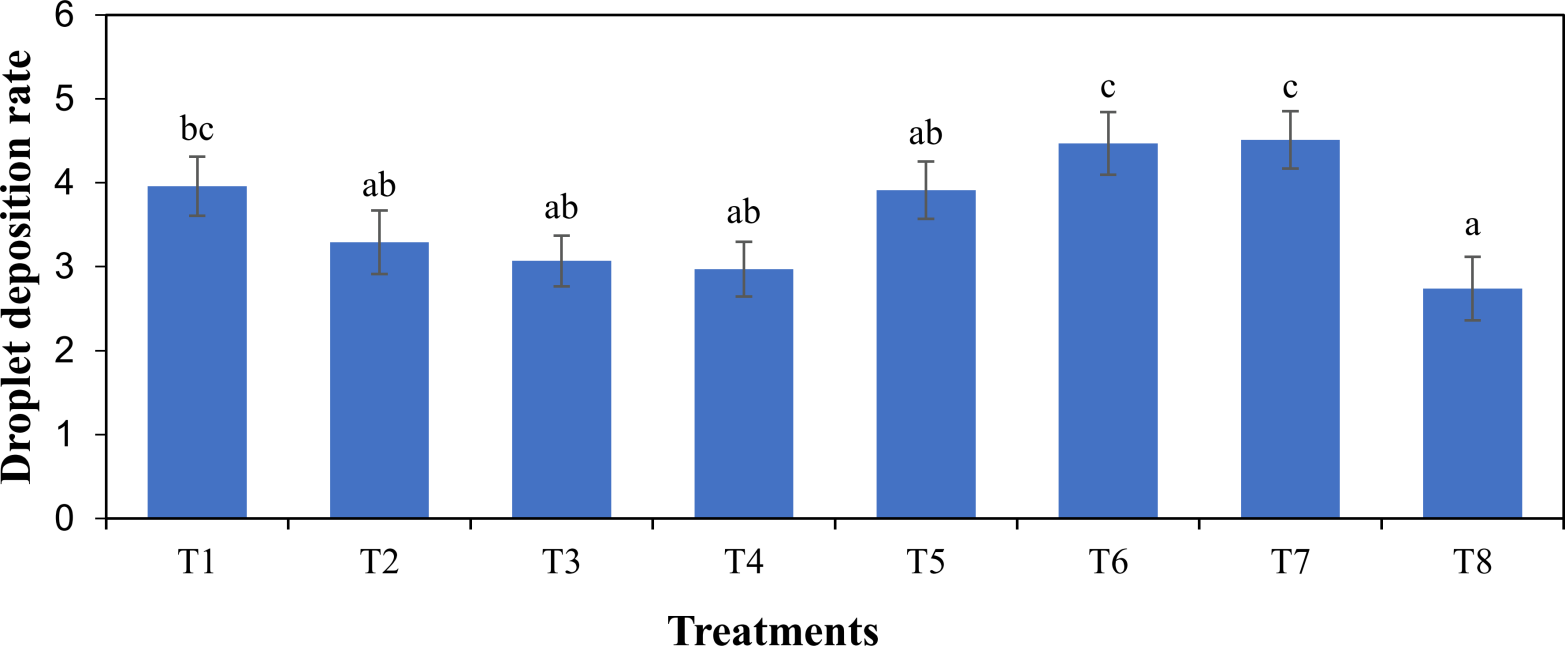
Average droplet deposition (μL/cm^2^). Means sharing similar letters are not significantly different at *p* > 0.05. T1 was 20% acetamiprid SP with nongjianfei, T2 was 20% acetamiprid SL with nongjianfei, T3 was 10% acetamiprid EC with nongjianfei, T4 was 40% acetamiprid WG with nongjianfei, T5 was 20% acetamiprid WP with nongjianfei, T6 was 5% acetamiprid ME with nongjianfei, T7 was water with nongjianfei, and T8 was water.

The enhanced physicochemical properties have a direct effect on the deposition process. There was a significant effect of the treatments (*F* = 3.77, *p* < 0.05) on the average droplet deposition of the solution. The average droplet deposition in the target area for all treatments is given in **Figure 10**. The droplet deposition in the target area showed that the combination of 5% acetamiprid ME with nongjianfei gave a higher droplet deposition (4.47 μL/cm^2^) than other formulations of acetamiprid. Similarly, 40% acetamiprid WG added with nongjianfei showed a lower deposition when compared with other formulations. The droplet deposition values for 5% acetamiprid ME with nongjianfei and 40% acetamiprid WG added with nongjianfei within the target area were 4.47 μL/cm^2^ and 2.97 μL/cm^2^, respectively. The adjuvant alone showed the highest droplet deposition (4.51 μL/cm^2^) when compared with all treatments within the target area. In case of water, the least droplet deposition was found for all tested treatments.

## 4 Discussion

The results indicated that all the formulations significantly improved the physicochemical properties, including density, viscosity, and surface tension. The physicochemical properties improved further when the formulations were added with adjuvants. The results are consistent with Zhao et al.’s (2022a) findings, which showed that adjuvants with the addition of pesticides notably enhanced the physical and chemical characteristics of spray dilutions, reduced spray droplet bounce, and enhanced the wetting and spreading abilities of spray dilutions. In addition, Meng et al. (2021) found that the adjuvants had the potential to greatly reduce surface tension and boost the spreading. Duan et al. (2022) reported that all the formulations give a lower density than water, and formulations showed higher viscosity and lower surface tension and significantly affected the chemical properties of the solution. The increase in viscosity decreases the droplet bounce and hence enhances the droplet deposition (Song et al., 2019). According to Preftakes et al. (2019), certain adjuvants have the ability to help in minimizing the number of pesticides, improve the effectiveness of pesticide control, make it easier to transport pesticide chemicals, and lower the amount of pollution that is released into the environment. The findings indicate that adding a standard adjuvant called nongjianfei to acetamiprid formulations has notable effects on reducing contact angle, increasing droplet size and coverage, and enhancing penetration for a specific type of UAV. The results were consistent with a prior study by Meng et al. (2022) who demonstrated that the common tank-mix adjuvant nongjianfei effectively decreases the contact angle of aqueous sprays. Although the adjuvants are commonly used in pesticides, their function and quality of spray is not always clear. The influence of adjuvants on retention, deposition, and translocation movement into the leaf and through the plants and their activity to control the target pest may be different for different pests and crops. The results showed that the formulations when added with adjuvants can significantly increase the deposition on the target surfaces. However, their mechanisms of how the adjuvants affect the deposition and increase the performance of different formulations using different methods of applications such as UAVs require further study.

In this study, the control efficiency of acetamiprid formulations with adjuvants was also investigated. The acetamiprid formulations in conjunction with adjuvants showed a higher mortality of cotton aphids compared to the control treatment. The higher mortality of aphids observed with the combination of adjuvants was attributed to several reasons. The addition of adjuvants improved the physicochemical properties of the solution particularly the combination of 5% acetamiprid and nongjianfei exhibited higher viscosity and reduced the surface tension, which increased the droplet spread and adhesion on the surface of leaves. This increased the contact and efficacy of pesticide against the cotton aphids. Moreover, the measurement of contact angle demonstrated that the addition of adjuvants had significantly reduced the contact angle that enhanced the wettability of the solution. The enhanced wettability provides better coverage on the leaf surface, which is critical for the effective control of pests. These results were consistent with the findings of Appah et al. (2020) and Chen et al. (2023). Furthermore, the findings of the present study are similar to the existing literature reported by Meng et al. (2018) who reported that by employing a suitable tank-mix adjuvant, the pesticide dosage may be decreased by 20% without compromising the control efficiency. The studies conducted in the past have proven to be feasible for low-volume spraying through UAVs in chemical application for controlling the pests and diseases (Chen et al., 2017, 2020). However, further optimizing and improving parameters is one area where further research can be done.

According to the ISO 22866:2005 standard, acceptable meteorological conditions for pesticide spraying include wind speeds below 3.3 m/s, temperatures between 5°C and 35°C, and relative humidity above 50% (Bourodimos et al., 2019). In the present study, the average wind speed was 2.5 m/s, which is within the standard’s limit. However, the temperature was 35.1°C and the relative humidity was 42.6%, slightly exceeding the ISO recommendations. It is important to note that the consistent application of the UAV’s technical specifications and the use of standardized methodology across all treatments helped to control these variables. While the temperature was slightly higher and the humidity was slightly lower, the overall trends observed in the efficacy of the formulations remain valid and provide valuable insights into their performance under slightly less ideal conditions, as in the real field setting, the meteorological conditions are not always ideal. Various studies have shown that droplet size measured in micrometers is the main component that affects drift and droplet deposition in pesticide spraying (Chen et al., 2023). When the droplet size of the pesticide solution is relatively small (less than 100 µm), the droplet tends to float longer in the air, and hence, it is more vulnerable to drift with the speed of wind (Liu et al., 2022). Ferguson et al. (2016) conducted a laboratory spray test to determine droplet coverage, droplet density, and size of droplets on oat plant canopies sprayed by different types of nozzles. Zhang et al. (2023) showed that the droplet deposition densities increased both on the upper and lower layers when the adjuvants were used in the aerial spraying tests. Therefore, the inclusion of an adjuvant can significantly enhance the deposition density of pesticide droplets. The findings of the present study also demonstrated that including adjuvants in the solution resulted in larger droplet sizes and increased coverage rates compared to the pesticide’s standalone impact. The acetamiprid 5% ME performed well in comparison to all other formulations when combined with adjuvants. These results were consistent with the findings of Liu et al. (2022) who found that adjuvants have the ability to modify the size and distribution of droplets within the crop canopy. Additionally, recent studies have also explored the dynamics of aerial spraying through UAVs. Yu et al. (2024) highlighted the effect of droplet deposition characteristics, which aligns with the findings of the current study on the impact of adjuvants on droplet deposition. Similarly, Qin and Chen (2023) have provided a comprehensive analysis of the research progress on droplet deposition and drift that is consistent with the results of optimizing the droplet characteristics and deposition by using appropriate formulations and adjuvants. Furthermore, the findings of the droplet coverage and the droplet density were consistent with the study conducted by Zhang et al. (2023) who demonstrated that the adjuvants can significantly enhance the droplet coverage by 71.47% and 41.55% in the upper and lower layer, respectively, and the droplet density by 71.91% and 98.45% in the upper and lower layer, respectively.

Pesticide spray drift is a major environmental issue resulting from the aerial application of UAVs and significantly impacts the efficacy and management of pesticides (Otto et al., 2015). We found that the wind speed had a significant impact on droplet drift. Different formulations showed different drift rates under different wind speeds. The differences in the meteorological conditions between T5 and T6, particularly wind speed, had a notable impact on the droplet drift. As stated, the higher speed in T5 (2.6–2.9 m/s) and T6 (2.9–3.3 m/s) has increased the droplet drift compared to the other treatments with a lower wind speed. Despite the higher wind speed, the combination of the 5% acetamiprid ME and nongjianfei demonstrated lower drift compared to other combinations, showing its efficacy under challenging conditions. On the other hand, 40% acetamiprid at a moderate wind speed of 1.2–2.1 m/s exhibited a higher drift rate. Furthermore, the findings indicated that all formulations and adjuvant combinations exhibited reduced drift compared to the control treatment, which solely contained water and the tracer. These findings were consistent with those of Grant et al. (2022) who showed that an increase in wind speed correlates with a greater spray drift of the solution, which significantly affects the droplet deposition. Additionally, the results align with prior research conducted by Preftakes et al. (2019) who found that the SC formulations gave 37% less drift than the WP formulations while drift was reduced 63% when the adjuvants were incorporated into formulations. Similar findings were reported by Wang et al. (2018b) who showed that the adjuvants can significantly reduce the droplet drift compared to water. Moreover, Brown and Giles (2018) found that different adjuvants could reduce the spray drift at different rates and that spray drift can be affected by the physical characteristics of the solution like climatic conditions, operational techniques, and droplet size distribution. Based on the previous results of the laboratory experiments, we found that the physicochemical properties of acetamiprid formulation were improved by adding adjuvants; in particular, 5% acetamiprid ME and nongjianfei showed lower surface tension than other tested formulations and adjuvants. This suggests that they can significantly reduce the droplet drift by lowering the surface tension. Similar findings were revealed by Oliveira et al. (2015) and Wang et al. (2024), which demonstrated that the addition of adjuvants reduced the droplet drift by improving the physicochemical properties, particularly reduced surface tension and increased viscosity. Further studies could investigate a wider range of pesticides and adjuvants to develop tailored solutions for enhanced spray efficiency, less droplet drift, and environmental safety.

For the selection of effective pesticides, it is essential that the spray dilutions should be effectively deposited on the leaves. Zhao et al. (2022b) reported that using appropriate adjuvants upon adding with pesticide formulations for UAVs in field circumstances can improve the deposition and distribution of pesticides on leaves, decrease the amount of pesticide needed, and enhance pesticide efficiency. In another study Zhao et al. (2022a) showed that using appropriate adjuvants with pesticide solution in UAV-based plant protection can greatly enhance spray dilution performance, boost pesticide dosage delivery efficiency, and enhance disease control. These adjuvants can help to enhance the effectiveness of insecticides and to minimize the amount of chemicals. Based on the results of the aerial application in the target area, it was found that the adjuvants can increase the deposition of pesticide formulation. Furthermore, the improved deposition rate as observed in the current study could lead to the reduction of chemical use by increasing the efficiency of pesticide via adding adjuvants and it also contributes to more sustainable agricultural practices. Further studies could be focused on the long-term effect of these formulations and adjuvants on crop health and ecosystem balance as well as the economic effect of these technologies for standard farming practices.

## 5 Conclusion

The findings of the present study indicate that adding adjuvants to acetamiprid formulations raised the viscosity of the formulations while decreasing their surface tension. The combination effect of formulations and adjuvants enhanced the efficiency of pesticide droplets by reducing the contact angle and boosting the spreading rate on leaf surfaces. The 5% acetamiprid microemulsion performed well compared to other formulations in terms of its physicochemical properties and efficiency in controlling pests. Furthermore, the nongjianfei was the most effective enhancer, significantly improving the effectiveness of all acetamiprid formulations when combined with it. The optimal qualities were demonstrated by the combination of 5% acetamiprid ME and nongjianfei solution, enhancing deposition features and increasing the mortality rate of cotton aphid. The findings indicate that the adjuvants can significantly affect acetamiprid formulations, resulting in a higher droplet size, coverage, and density. The results of current studies provide a solid foundation for the development of precision agriculture technology that includes the UAV-based spraying system.

## Data availability statement

The original contributions presented in the study are included in the article/supplementary material. Further inquiries can be directed to the corresponding author.

## Author contributions

MZ: Conceptualization, Investigation, Software, Writing – original draft. HL: Software, Writing – original draft, Investigation, Project administration. GY: Data curation, Investigation, Software, Writing – original draft. HR: Data curation, Investigation, Project administration, Writing – original draft. YL: Software, Writing – original draft, Investigation. MA: Formal analysis, Writing – original draft, Software. ZD: Investigation, Writing – original draft, Software. XH: Funding acquisition, Project administration, Resources, Supervision, Validation, Visualization, Writing – review & editing, Conceptualization, Writing – original draft.
